# The Effect of Multiple Autoclave Sterilization on the Cyclic Fatigue of Three Heat-Treated Nickel-Titanium Rotary Files: EdgeFile X7, Vortex Blue, and TRUShape

**DOI:** 10.1155/2020/8826069

**Published:** 2020-12-14

**Authors:** Reham Sharroufna, Mohammed Mashyakhy

**Affiliations:** ^1^Private Dental Practice, Riyadh, Saudi Arabia; ^2^Department of Restorative Dental Sciences, College of Dentistry, Jazan University, Jazan, Saudi Arabia

## Abstract

**Objective:**

The purpose of this study was to evaluate the effect of repeated autoclave sterilization on the cyclic fatigue resistance of heat-treated NiTi rotary endodontic instruments.

**Materials and Methods:**

Three NiTi rotary endodontic instruments (EdgeFile X7, EFX7 0.30/0.4; Vortex Blue, VB 0.30/0.4; and TRUShape, TS 0.30/0.6) were selected. Each group (*n* = 24 each) was divided into 2 subgroups (*n* = 12 each): sterilized instruments and nonsterilized instruments. The sterilized instruments were subjected to 10 cycles of autoclave sterilization. Twelve instruments from each different subgroups were tested for cyclic fatigue resistance, and the number of cycles to failure (NCF) was calculated. Means and standard deviations were calculated for each group, and data were statistically analyzed using the SPSS program (*P* < 0.05).

**Results:**

Sterilized and nonsterilized EFX7 files showed the highest NCF compared with other file subgroups. Comparing the results between sterilized and nonsterilized instruments for each type of files, there was a statistically significant difference (*P* < 0.05) only between sterilized and nonsterilized EFX7 files (1198 versus 755 NCF). The other instruments did not show significant differences (*P* > 0.05) in the mean NCF as a result of sterilization cycles (VB, 606 versus 568 NCF; TS, 487 versus 442 NCF).

**Conclusion:**

Repeated cycles of autoclave sterilization increased the NCF of the new heat-treated files, with EFX7 showing statistically significant superior results compared with other files tested.

## 1. Introduction

Chemomechanical cleaning and shaping is the most critical step of root canal treatment (RCT) [1–4]. This important step, with the help of nickel-titanium (NiTi) rotary endodontic files, becomes faster and more predictable; however, there is an uneventful risk of instrument separation, and it is a well-reported challenge in endodontic literature [5–8]. Despite the outstanding properties of recent NiTi instruments, they still can be separated due to cyclic or flexural fatigue [9–13]. Cyclic fatigue is reported to occur as a result of repeated bending of the instruments in curved canals that lead to deformation and stress within the instruments, which end up with fracture due to complete tension-compression cycles [14]. Manufacturers developed thermomechanical processing by adjusting the transformation temperatures of the NiTi alloys to improve the fatigue resistance to overcome instrument fracture [15]. Thermal treatment of NiTi alloy has shown favorable effects on mechanical and physical properties of NiTi files [16]. In clinical practice settings, NiTi rotary files might be used many times, and the repeated sterilization process by autoclave is necessary to prevent cross-contamination [17, 18]. A 2020 systematic review and meta-analysis study found that autoclave was the most effective method to sterilize endodontic instruments [19].

Many studies had investigated the sterilization using autoclave effects on the physical properties of NiTi rotary files and reported contrasting results. Some showed that repeated sterilization badly affects the files and cause fractures [20–24]. While other studies reported that repeated cycles of heat sterilization did not cause the failure of NiTi files [25, 26]. Plotino et al. [27] showed that repeated sterilization only significantly increases the cyclic fatigue resistance of one file (K3XF) out of four groups that were tested, where their properties were not influenced. Also, Zhao et al. [18] observed an extension of the cyclic fatigue life of HyFlex CM and K3XF compared with three other tested groups. A recent review [19] showed that hot sterilization by autoclave generally did not alter the physical and mechanical properties of most NiTi endodontic files. Studies seem to be inconsistent and showed different results in different types of NiTi endodontic rotary files. Many heat-treated NiTi endodontic files are found in the market with a variety of metallurgic properties. Vortex Blue (VB; Dentsply Tulsa, Tulsa, OK, the USA) is a NiTi rotary instrument manufactured by a proprietary thermomechanical process to improve fatigue resistance, cutting efficiency, flexibility, and canal centering capability [28]. It has been reported that Vortex Blue has a significant cyclic fatigue resistance compared with similar sizes of NiTi files that are made of M-wire [29]. EdgeFile X7 instruments (Edge Endo; Albuquerque, New Mexico, United States) is a NiTi file which has a constant taper, manufactured by a process called “FireWire™,” which potentially increases the flexibility and resistance to fatigue and reduces the shape memory effect inherent of NiTi instruments [30]. A recent study [31] showed that EdgeFile instruments have higher fatigue resistance than VB files. TRUShape (TS; Dentsply Tulsa Dental Specialties, Tulsa, OK) is a novel heat-treated NiTi file where the heat treatment is applied after flutes are ground into blanks; it is characterized by an S-curve and a taper of 0.06 in the apical 2 mm. TS file has recently been introduced in the market and not well studied [32].

The aim of this study, therefore, was to evaluate the effect of multiple autoclave sterilization on the cyclic fatigue of three types of thermally treated NiTi files (Vortex Blue, EdgeFile X7, and TRUShape). The null hypothesis is that no significant difference could be found between the three types of heat-treated NiTi endodontic files in terms of cyclic fatigue after multiple autoclave sterilization.

## 2. Materials and Methods

Three types of NiTi rotary endodontic heat-treated instruments were selected: EdgeFile X7 (EFX7 group) 0.30 mm tip diameter and 0.4 taper (EdgeEndo, Albuquerque, NM, USA), Vortex Blue (VB group) 0.30 mm tip diameter and 0.4 taper (Dentsply Tulsa, Tulsa, OK, the USA), and TRUshape (TS group) 0.30 mm tip diameter and 0.6 taper (Dentsply Tulsa, Tulsa, OK, the USA). All files had the same length of 25 mm. Twenty-four instruments from each type were collected (total = 72 files). Each group was then divided into 2 subgroups (*n* = 12 each), sterilized (S) and nonsterilized (NS) instruments. The sterilized instruments were subjected to autoclave sterilization (10 cycles) at a temperature of 134°C for 35 minutes for each cycle (time included 20 minutes for sterilization and 15 minutes for drying).

For testing, a previously described methodology was followed [27]. In brief, the device consisted of mobile plastic support connected to the mainframe of the device. This plastic support was used to hold the handpiece and metal block containing the artificial canals. Each instrument was tested in a simulated root canal with a 60° angle of curvature and 5 mm radius of curvature. The center of the curvature was located 5 mm from the tip of the file, and the curved segment of the canal measured about 5 mm in length.

A contra-angle (6 : 1) handpiece (Sirona, Bensheim, Germany) powered by a torque-controlled electric motor (E3; Dentsply Tulsa, Tulsa, OK) was used to rotate the instruments at a constant speed of 300 rpm until their fracture occurred. The number of cycles to failure (NCF) was calculated by multiplying the time to fracture by the speed of the handpiece. The data of NCF were analyzed using one-way analysis of variance (ANOVA) and Tukey's post hoc tests. The level of significance was set at *P* < 0.05 for all tests.

## 3. Results

As shown in Table 1, higher values of NCF were noticed in sterilized groups compared to nonsterilized groups with the highest value 1198.76 ± 255.56 for the EFX7(S) group and lowest value 442.22 ± 80.92 for the TS(NS) group. There was a highly statistically significant difference (*P* < 0.001) between sterilized and nonsterilized EFX7 instruments. However, no differences were found between sterilized and nonsterilized Vortex Blue as well as True Shape instruments (*P* > 0.05). Generally (regardless of sterilized or not), the EFX7 group had the highest cyclic fatigue resistance followed by the VB group and the TS group.

Based on Tukey's post hoc test (Table 2) for multiple comparisons, there were significant differences (*P* < 0.001) between the EFX7(S) group and all other groups. Also, there were significant differences between the EFX7(NS) group and TS(S) and TS(NS) groups. However, no significant differences were found between all other instrument groups (*P* > 0.05).

## 4. Discussion

Dental practitioners usually use NiTi endodontic files repeatedly for practical and economic reasons, which are accompanied by many cycles of autoclave sterilization [18]. Manufacturers promote a single use of NiTi files for fracture safety; however, we chose to perform our study doing 10 cycles by autoclave as other previous studies [27, 33] to assess the effect of repeated sterilization trying to mimic a clinical use. The superior properties of NiTi alloy are highly dependent on the thermomechanical processing of the manufactured product [34], and additional heat of the instrument by autoclave sterilization may increase its mechanical and physical properties as suggested by Yahata et al. [16]. Up to our best knowledge, there are no available studies in the literature evaluating the cyclic fatigue resistance of VB, EFX7, and TS instruments after repeated cycles of autoclave sterilization. So, in this in vitro study, we investigated the effect of repeated cycles of autoclave sterilization on cyclic fatigue of new heat-treated files that are commercially available in the market. Although lab testing has some limitations, the results could be of practical value [35].

The results of the current investigation did not support the null hypothesis as the number of cyclic to fracture was higher in sterilized compared with unsterilized endodontic files. Interestingly, the main finding is that sterilized NiTi instrument groups had more cyclic fatigue resistance compared with nonsterilized ones, with only EFX7 showing a highly statistically significant difference. In addition, VB files performed better than the TS ones in both sterilized and nonsterilized groups. The results of NS groups are consistent with a previous observation, where EdgeFile instruments had higher fatigue resistance compared with VB files when subjected to different temperature settings [31]. In another study, however, EFX7 did not show superior cyclic fatigue resistance and was similar to HyFlex and MTwo but significantly lower than the ProDesign logic NiTi instrument for the same file size [36]. In regard to TS and VB files, a study showed no significant difference between TS and VB at 20°C, but when the temperature rose to body temperature, VB showed higher cyclic fatigue resistance than TS [37]. Also, other studies reported that TS files had less fatigue resistance compared with other files tested [38, 39].

Many studies evaluated the effect of sterilization on mechanical properties and performance of NiTi endodontic instruments with contradicting results [40–52]. Repeated cycles of sterilization of ProFile instrument [25] and LightSpeed instruments [41] did not increase their resistance to cyclic fatigue, while in a more recent study [42], ProFile NiTi showed higher NCF when exposed to autoclave sterilization. These studies have been done on traditional NiTi endodontic files, whereas only a few ones investigated the influence of a repeated cycle of autoclave sterilization on cyclic fatigues of new heat-treated NiTi instruments [18, 27, 33, 53]. Our results showed clearly that the repeated cycle of autoclave sterilization improved the mechanical properties of the tested files where more NCF is needed. This finding is in agreement with Zhao et al.'s study [18], where autoclave sterilization increased the cyclic fatigue of HyFlex CM and K3XF, which are made of thermally treated alloy. Also, Plotino et al. [27] tested K3 XF files, produced with an innovative heat-treated NiTi alloy, and showed a statistically significant increase in the CNF after 10 cycles of autoclave sterilization. In contrast, another study [33] found that repeated sterilization did not significantly affect 3 of the 4 groups of NiTi instruments tested (GTX 20.04, GTX 20.06, Twisted File 25.04). However, another study [53] evaluated (PTU, K3X, TFA, and EDM) NiTi files and reported that sterilization did not influence the cyclic fatigue significantly.

Our study showed generally that all files tested (regardless of sterilized or not) showed higher cyclic fatigue resistance with the EFX7group having the highest NCF followed by the VB group and the TS group. It seems that the new generation of heat-treated NiTi files tends to have better metallurgic properties, and the repeated heat of sterilization improved their resistance to cyclic fatigue. In addition, interestingly, the EFX7 nonsterilized group showed significantly higher CFR than all other file subgroups (VB and TS), and that may be related to the “WireFire™” technology used and its special design. More studies are highly recommended in a body temperature simulation to mimic the clinical situation for more reliable results which is one of the main limitations of our study.

## 5. Conclusion

Within the limitations of this in vitro study, it can be concluded that repeated cycles of autoclave sterilization increased the NCF of the new heat-treated files tested, with EFX7 made by FireWire™ technology showing statistically significant superior results.

## Figures and Tables

**Table 1:** Mean ± SD and mean differences between sterilized and nonsterilized instruments.

Type	*N*	Mean ± SD	Mean difference	95% CI of difference		*P*
				Lower	Upper	
EdgeFile X7						
Sterilized	12	1198.8 ± 255.6	443.0	246.9	639.0	*P* < 0.001
Nonsterilized	12	755.8 ± 175.4				
Vortex Blue						
Sterilized	12	606.1 ± 145.1	37.4	-158.7	233.5	0.993
Nonsterilized	12	568.7 ± 174.6				
TRUShape						
Sterilized	12	487.8 ± 80.7	45.5	-150.6	241.6	0.983
Nonsterilized	12	442.2 ± 80.9				

Significance level at *P* < 0.05.

**Table 2:** Tukey's post hoc test for multiple comparisons between different groups.

Type		Mean difference	95% confidence interval		*P*
			Lower	Upper	
EdgeFile (S)	Vortex Blue (NS)	630.0	433.9	826.1	<0.001
	Vortex Blue (S)	592.6	396.5	788.7	<0.001
	TRUShape (NS)	756.5	560.5	952.6	<0.001
	TRUShape (S)	711.0	514.9	907.1	<0.001
EdgeFile (NS)	Vortex Blue (NS)	187.1	-9.0	383.2	0.070
	Vortex Blue (S)	149.7	-46.4	345.8	0.234
	TRUShape (NS)	313.6	117.5	509.7	<0.001
	TRUShape (S)	268.1	72.0	464.1	0.002
Vortex Blue (S)	TRUShape (NS)	163.9	-32.2	360.0	0.153
	TRUShape (S)	118.4	-77.7	314.5	0.491
Vortex Blue (NS)	TRUShape (NS)	126.5	-69.6	322.6	0.415
	TRUShape (S)	81.0	-115.1	277.1	0.829

Significance level at *P* < 0.05.

## Data Availability

Data are available upon request from the corresponding author.
